# Radio-Therapy

**Published:** 1905-05-20

**Authors:** 


### PROGRESS IN MEDICINE AND SURGERY.

### RADIO-THERAPY.

There are few more disfiguring and disheartening affections than the extensive superficial nævi. Repeated surgical treatment is apt too often to have very indifferent results. It is to some purpose then that we find physical methods of treatment advanced for the relief of these pitiable conditions. Either $x$-rays or radium may be used with success. The good result, however, can only be obtained after somewhat severe reactions. Levack[1] has repeated Jatassey's experiment with the $x$-rays. He has found that after causing sufficient reaction to produce a raw surface had been induced, though the healing was slow, yet the results in three cases compared favourably with older methods, such as scarification, caustics, and actual cautery. The scar, he says, differs little from the normal skin, being of the same colour and quite as pliant as its surroundings. Even deeper nævi may be attacked, though these require more persistent care than the superficial ones.

Hartigan[2] has used radium for a port-wine mark disfiguring the left cheek, eyelid, upper lip, and nose of a woman aged 26. Ten milligrams of radium were used in an adjustable vulcanite capsule, and applications of 10 mins. were made until vesication was produced. On healing, white areas were left.

The risk of incurring some form of dermatitis has rendered the use of the $x$-ray machines a matter of considerable inconvenience and even danger to the workers. Cases have even been recorded in which a carcinomatous degeneration has appeared on the hands of those affected with the chronic dermatitis. That the $x$-rays are directly responsible for this may be doubted, and Milton Franklin[3] states clearly that the well-known cases of Dr. Blacker and Clarence Dalby are of "indirect causation," while Dr. Hall-Edwards[4] states that we have not as yet had placed before us the true and authentic history of a single case of $x$-ray dermatitis developing into cancer. That the patient should be in any danger of having carcinoma induced as a result of continued application of the rays seems to be a very remote contingency, yet Norman Walker[5] mentions such possibility in the case of lupus, even quoting an instance where the development was watched. It is of great moment, however, to compare the dangers of the new methods with those of the older, and his article is of interest in this particular. It is a well-recognised fact that carcinoma is apt to develop upon lupus or the scars of lupus. Walker states that 31 per cent. of the recorded cases were in patients under 40 years of age, and in 90 per cent. cancer developed on the face. He holds that in all cases it is more probable that the cancer develops from the scar-tissue, and not from the lupus-tissue proper. He thinks that there may be reason for accepting Cohnheim's theory of buried epithelium being the point of origin. In sections of lupus it is not uncommon to find long processes of epithelium ramifying in the cellular tissue resembling closely epithelioma. That these processes should be nipped off by encroaching scar tissue and become to all intents and purposes Cohnheim's "rests," capable at any time of malignant development, seems plausible; this is his explanation of the complication of lupus by carcinoma. It is not impossible that the old method of scraping may lead to the condition mentioned, apart from the possibility of spreading the general infection of tuberculosis throughout the body. Contrasting the epithelioma found in $x$-ray workers with that found in rayed lupus, he draws attention to the fact that the former is of a hard warty type, which if not promptly treated grows with frightful rapidity, involves glands and requires extensive operation, which may prove ineffectual. In lupus, however, the condition is often papillomatous, but has not the hyperkeratosis of the $x$-ray cancer; metastasis is quite exceptional and death is due to local extension. Walker has had four cases of carcinoma developing on lupus, one of which has not been rayed. Of the remaining three one only he considers to have been due to the rays. In none had "burns" been produced. He states that perseverance in the application of the rays, a course requiring at first some courage, may directly cure the condition which it has indirectly caused.

Among the other deleterious results of $x$-rays may be placed inflammation of the optic nerve,[6] conjunctivitis,[7] and cellulitis of the upper lid,[8] periostitis[9] and paralysis with neuralgic pain,[10] headache and vertigo after irradiation of the head.[11] Sterility in $x$-ray workers has been observed, by Tilden Brown and Osgood[12] in 16 cases, though how this is produced is as yet uncertain, as in one case which came under their notice the individual had active spermatozoa in spite of having attended frequently at $x$-ray séances. It may be that repeated and prolonged exposures are necessary to produce this effect.

The $x$-rays are finding a useful place in the treatment of ringworm. Hitherto the treatment has been considered so fraught with the danger of producing a cicatricial and permanent alopecia that it has only been used with the greatest caution. Sabouraud[13] has brought forward a more perfect method of measuring the dosage of the rays, and has used this method with sufficiently successful results as to establish its use, from the points of view of rapidity and completeness as well as from that of diminished cost. He has used it at the Lailler School in Paris. In 15 days he is able to produce a depilation of the area treated, and finds that the hair grows again in a healthy condition 10 weeks after the beginning of the treatment. Contagion he finds to disappear 25 days after the treatment is commenced. Radio-dermatitis must be avoided absolutely, and he found that none of the ordinary methods of measuring the rays proved efficient in preventing this. His dosage is up to five units of Holzknecht. As Holzknecht's pastilles are expensive and their colour accentuates after the rays are removed, Sabouraud used paper painted with an emulsion of platino-cyanide of barium in amyl-acetate collodium. This changes colour under the rays, and when the colour has reached a fixed water-colour tint corresponding to five units of Holzknecht the dose is sufficient. The comparison must be made rapidly, as light fades the colour. The emulsion is not so sensitive to the rays as the pastilles, and it should be 8 cms. from the anti-kathode, while the patient is 15 cms. distant. There is no danger so long as the paper at 8 cms. has not reached the tint of limit.

Colcott Fox,[14] in praising the treatment, states that "the hairs can be made to fall without serious damage to the scalp, and are replaced after an interval of some weeks by a healthy new growth. From every point of view this must become the treatment of the future, where the rays are obtainable. It is painless, comparatively rapid, and economical. All danger of infection to others is rapidly removed, and the only drawback is a somewhat prolonged period of more or less baldness.

The treatment of the "blood diseases" continues to give interesting results. The symptoms are affected and for the most part the patient is greatly improved, though not always permanently; and in spite of the disappearance of the distressing symptoms, in some cases the patient has succumbed. Typical cases are published by Joachim and Kurpjuweit.[15] In their hands a case of anæmia splenica did not improve, but there was marked improvement in one case of leukæmia, and some improvement in a second. In the first case the blood-count showed leucocytes 693,000, erythrocytes 2,500,000; after three and a half months of treatment intermittently with the $x$-rays the leucocytes were 6,300, and the erythrocytes 3,400,000, while the general condition was excellent; In the second case, one of chronic lymphæmia with enlargement of the glands in the axilla, under the jaw, and in the inguinal region, with liver and spleen enlarged, the blood-examination showed whites 385,000, 98-90 per cent. lymphocytes, reds 3,200,000. After $x$-rays treatment the lymph glands nearly disappeared, and the spleen became much smaller, the white cells decreased in number, but they were still lymphatic in character.

[1] Scott. Med. and Surg. Jour., July, 1904. [2] Amer. Jour. Med. Sci., November, 1904. [3] Archiv Roent. Rays, January, 1905. [4] Med. Electrol. and Radiol., February, 1905. [5] Scott. Med. and Surg. Rev., July, 1905. [6] Archiv Roent. Rays, January, 1905. [7] Ibid. [8] Ibid. [9] Ibid. [10] Ibid. [11] Ibid. [12] Ibid., March, 1905. [13] Annales de Derm. et de Syph., July, 1904; Brit. Jour. of Derm., February, 1905; Treatment, December, 1904. [14] Practitioner, April, 1905. [15] Brit. Med. Jour. Epit., March 4, 1905.

(*To be continued.*)

